# Antenatal Consultation and Postnatal Stress in Mothers of Preterm Neonates (A Two-Center Observational Case–Control Study)

**DOI:** 10.3389/fped.2017.00275

**Published:** 2017-12-20

**Authors:** Elisabeth Pichler-Stachl, Nariae Baik-Schneditz, Bernhard Schwaberger, Berndt Urlesberger, Gerhard Pichler, Po-Yin Cheung, Georg M. Schmölzer

**Affiliations:** ^1^Division of Neonatology, Department of Paediatrics, Medical University of Graz, Graz, Austria; ^2^Department of Pediatrics, University of Alberta, Edmonton, AB, Canada; ^3^Centre for the Studies of Asphyxia and Resuscitation, Royal Alexandra Hospital, Edmonton, AB, Canada

**Keywords:** neonatologist, antenatal consultation, preterm birth, neonatal intensive care unit, maternal stress

## Abstract

**Background:**

During antenatal consultation of women hospitalized for preterm labor, information of possible adverse outcomes is provided. This may however create additional maternal stress and raise some ethical concerns.

**Objective:**

The aim of the present study was to evaluate the influence of antenatal consultation by a neonatologist on maternal stress after delivery of a preterm infant admitted to NICU.

**Methods:**

In this study, secondary outcome parameters of a prospective two-center pilot observational study were analyzed. Mothers of preterm neonates < 36 weeks of gestation admitted at two tertiary-level Neonatal-Intensive-Care-Units (NICU) were included. Maternal stress was assessed with the Parental-Stress-Scale:NICU (PSS:NICU) within 72 h after birth. PSS:NICU measures three scales: “relationship and parental role,” “sights and sounds,” and “baby looks and behaves.” Maternal sociodemographic data were collected by questionnaire administered at the same time. Mothers who received antenatal neonatal consultation were matched for gestational age and compared to mothers who had no antenatal consultation by a neonatologist.

**Results:**

A total of 46 mothers of preterm neonates were included, 23 mothers in each group. There was no significant difference in sociodemographic data between the two groups regarding neonates and mothers. There were no significant differences between the two groups regarding stress scales of “sights and sounds” (2.00 ± 0.76 versus 2.19 ± 0.79; *p* = 0.402), “looks and behaves” (2.55 ± 0.90 versus 2.48 ± 0.94; *p* = 0.732) and “relationship and parental role” (3.28 ± 1.23 versus 3.46 ± 1.07; *p* = 0.517).

**Conclusion:**

Our study demonstrated that antenatal consultation by a neonatologist had no substantial influence on postnatal maternal stress in mothers of preterm neonates admitted to the NICU.

## Introduction

Already 70 years ago data suggested that maternal emotional stress during pregnancy might affect the fetus (1). Further, more than 50 years ago several studies demonstrated an association of maternal experiences and emotional stress during pregnancy and the health of the infant resulting in an impaired level of motor development (2). Many studies focused on maternal and paternal stress were published in the last decades. A meta-analysis recently reported that parents of preterm infants experienced more stress than parents of term infants (3). The differences in stress level between parents of preterm and term neonates are described to be small (4). However, neurobehavioral development of these sick neonates depends on parental stress and preterm birth is associated with altered parental mental health and family wellbeing after birth (3, 5, 6).

Low gestational age and birth weight are main factors affecting parental stress (2, 5–7). However, stress is determined by many other factors. Recently, we have demonstrated that the level of maternal stress after preterm birth increases with the length of antenatal hospital stay (8). This study suggested that antenatal intra-hospital management might have an influence on postnatal maternal stress. After hospital admission mothers with imminent preterm birth are generally informed by obstetricians about the imminent obstetrical management and by pediatricians/neonatologists about management and concerns about prematurity. A recent study reported that antenatal consultation by a neonatologist is perceived by women hospitalized for preterm labor as a source of stress and reassurance (9).

Different methods have been described to assess psychosocial stress such as physiological changes of heart rate, blood pressure, and cortisol level (10). Alternatively, stress may be assessed by using validated questionnaires such as the Trier Social Stress Test (TSST) (11), the Parenting Stress Index (PSI) (12), and the Parental Stressor Scale: Neonatal Intensive Care Unit (PSS:NICU) (13–16).

There are no data available, if antenatal consultation by a neonatologist has an impact on stress level of mothers after birth of premature infant admitted to NICU. The aim of the present study was therefore to evaluate the influence of antenatal consultation by a neonatologist for imminent premature birth on maternal stress level after birth of a premature neonate admitted to NICU. We hypothesized that antenatal consultation by a neonatologist for premature birth increases stress level due to information that is not only reassuring but also includes information of possible adverse outcome.

## Materials and Methods

The present observational case–control study is part of a two-center observational study at the Royal Alexandra Hospital of Edmonton/Canada and the Department of Pediatrics, Medical University of Graz/Austria (8). Secondary outcome parameters were analyzed. This study was carried out in accordance with the recommendations of Regional Committees on Biomedical Research Ethics with written informed consent from all subjects. All subjects gave written informed consent in accordance with the Declaration of Helsinki. The protocol was approved by the (i) The Royal Alexandra Hospital Research Committee and Health Ethics Research Board, University of Alberta and (ii) Ethikkommission, Medizinische Universität Graz.

Mothers of preterm infants born at < 36 weeks of gestation admitted to the NICUs were included. Exclusion criteria were neonatal congenital malformation and or chromosomal anomalies. Information was collected from data sources including parental questionnaire and accessing hospital charts. Mothers with antenatal consultation by a neonatologist were compared to mothers without antenatal consultation by a neonatologist. Mothers were matched for gestational age (±1 week) of their preterm babies.

The parental stress scale: neonatal intensive care unit (PSS:NICU) questionnaire was given to mothers of preterm infants within 72 h after birth and were asked to complete it within 24 h. The PSS:NICU questionnaire measures parental stress in three different scales: “relationship and parental role,” “sights and sounds,” and “baby looks and behaves” (14).

In the subscale “relationship and role” mothers are asked how they are affected by their relationship to the neonate and their role at the NICU, “sights and sounds” how mothers are affected by the visual and acoustic surrounding and, “babies looks and behaves” how mothers are affected by the neonate’s looks and behavior.

Maternal sociodemographic data were collected and the infant’s medical chart was accessed to record time of admission of the mother, time of birth, gestational age, birth weight, gender, need of respiratory support (nasal CPAP or mechanical ventilation), hemodynamic treatment (e.g., catecholamines), or the presence of cerebral injury (defined as intraventricular hemorrhage of any grade). The study was designed as a pilot study and therefore a convenient representative sample was used (17).

Differences between groups were analyzed using a Student’s *t*-test or Mann–Whitney *U* test for continuous parametric and non-parametric variables, respectively. To analyze differences in categorical variables Chi Square test and, when appropriate, Fisher’s Exact test were used. Continuous data are presented as median (interquartile range) or mean (SD). A *p*-value < 0.05 was considered significant.

## Results

**Figure 1:**
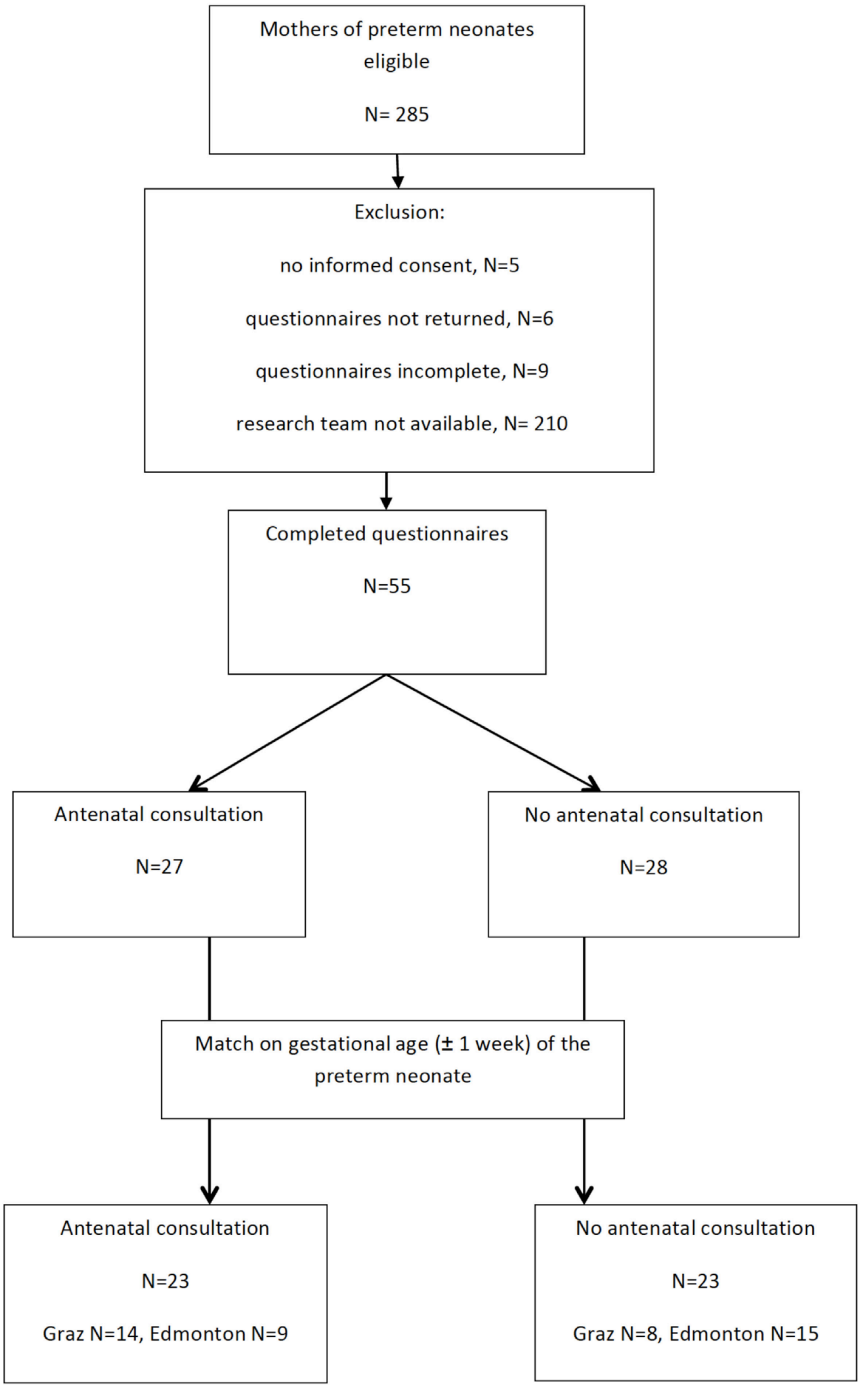
Mothers of preterm neonates included in this observational study.

During the study period, 155 preterm infants in Edmonton and 130 in Graz were eligible (Figure 1). Twenty-three mothers of preterm infants with antenatal consultation by a neonatologist were matched for gestational age to 23 mothers of preterm infants without antenatal consultation by a neonatologist. Median gestational age of preterm infants with and without antenatal consultation was 31 (27–34) weeks. Indications for preterm birth in mothers with antenatal consultation were spontaneous preterm labor (*n* = 11), premature preterm rupture of membrane (*n* = 4), eclampsia (*n* = 3), and others (*n* = 5). Indications for preterm birth in mothers without antenatal consultation were spontaneous preterm labor (*n* = 9), premature preterm rupture of membrane (*n* = 5), eclampsia (*n* = 4), and others (*n* = 5). There were no significant differences in maternal and neonatal demographic and clinical data between the two groups (Table 1).

**Table 1:** Demographic data of mothers and preterm neonates with and without antenatal neonatal consultation.

	Consultation	No consultation	*p-*Value
Maternal data	*n* = 23	*n* = 23	

Maternal age, years	30 ± 5	29 ± 4	0.228
Length of antenatal stay, days	1 (1–35)	2 (1–31)	0.520
Previous pregnancies, *n*	16	20	0.284
Previous life births, *n*	16	19	0.490
Previous premature births, *n*	6	7	0.650

Neonatal data	*n* = 23	*n* = 23	

Gestational age, weeks	31.0 ± 3.6	31.0 ± 3.7	0,817
Birth weight, g	1,568 ± 613	1,693 ± 684	0.173
Cesarean section, *n*	8	11	0.189
Male, *n*	10	13	0.207
Respiratory support, *n*	16	19	0.174
Hemodynamic support, *n*	7	4	0.490
Intraventricular hemorrhage, *n*	7	5	0.526

There were no significant differences in the stress level between both groups regarding the stress scales of “sights and sounds,” “looks and behaves,” and “relationship and parental role” assessed with the PSS:NICU questionnaire (Table 2).

**Table 2:** PSS:NICU results of mothers of preterm neonates with and without antenatal neonatal consultation.

	Consultation	No consultation	*p-*Value
Sights and sounds	2.00 ± 0.76	2.19 ± 0.79	0.402
Looks and behaves	2.55 ± 0.90	2.48 ± 0.94	0.732
Relationship and parental role	3.28 ± 1.23	3.46 ± 1.07	0.517

Moreover, there were no significant differences between the two centers in stress scales: “sights and sounds” (2.25 ± 0.78 versus 1.89 ± 0.72; *p* = 0.08) “looks and behaves” (2.68 ± 0.94 versus 2.42 ± 0.91; *p* = 0.29) and “relationship and parental role” (3.31 ± 1.17 versus 3.37 ± 1.16; *p* = 0.85).

## Discussion

This is the first study to compare stress in mothers of preterm neonates admitted to NICU with and without antenatal consultation by a neonatologist. Overall, we found no substantial association between antenatal consultation by a neonatologist and postnatal maternal stress. The stress level of mothers of preterm neonates admitted to NICU was within the range of formerly published studies (3, 8). Compared to published stress levels of term neonates the present stress levels in preterm neonates were elevated (3).

Women hospitalized for preterm labor and interviewed for their perspectives of antenatal consultation by a neonatologist expected the consultation as a stressor as well as a source of reassurance (9). During antenatal consultation, neonatologists are mandated to discuss potential postnatal medical management, prognosis, and possible adverse outcome of premature birth. In particular, information about short- and/or long-term adverse outcomes could act as a maternal stressor influencing postnatal stress levels. In a recent study, most women at risk of preterm delivery receiving antenatal consultation by a neonatologist reported a positive experience (18). Miquel-Verges et al. reported that mothers of neonates with known congenital anomalies want specific and realistic information during antenatal consultation (4). They also described that receiving conflicting information increased anxiety and decreased confidence. In comparison, our study suggests that information about adverse outcomes does not increase maternal stress once preterm neonates are admitted to the NICU. In the present study, antenatal neonatal consultations were performed by experienced neonatologists aiming to provide not only information of adverse outcomes but also reassurance and support. The latter might have caused the lack of increased stress after antenatal consultation (9). Limitations include that individual content of each consultation and the number of different neonatologists were not recorded. Another limitation is that the negative results might be due to the small sample size. However, due to the similar results in the subscales of the PSS:NICU, when both groups are compared, a substantial influence of antenatal neonatal consultation on maternal stress after delivery of a preterm infant admitted to NICU can be ruled out. A strength of our study was the two-center approach to compare maternal stress after preterm birth (Canada/Austria).

## Conclusion

Antenatal neonatal consultation in women hospitalized for imminent preterm birth does not substantially influence maternal stress after preterm birth. Information of possible adverse outcome and reassurance seems to balance maternal stress after preterm birth. However, maternal stress levels after preterm birth are increased after NICU admission when compared to maternal stress levels after term deliveries and no NICU admission. New strategies for antenatal consultation need to be developed to reduce maternal stress after preterm birth.

## Ethics Statement

This study was carried out in accordance with the recommendations of Regional Committees on Biomedical Research Ethics with written informed consent from all subjects. All subjects gave written informed consent in accordance with the Declaration of Helsinki. The protocol was approved by the (i) The Royal Alexandra Hospital Research Committee and Health Ethics Research Board, University of Alberta and (ii) Ethikkommission, Medizinische Universität Graz.

## Author Contributions

Conception and design; analysis and interpretation of the data: EP-S, GP, BU, P-YC, and GS. Collection and assembly of data: EP-S, GP, NB-S, BS, BU, and GS. Drafting of the article: EP-S, GP, and GS. Critical revision of the article for important intellectual content; final approval of the article: EP-S, GP, NB-S, BS, BU, P-YC, and GS.

## Conflict of Interest Statement

The authors have no financial relationships relevant to this article to disclose. No current funding source for this study. The funder had no role in study design, data collection and analysis, decision to publish, or preparation of the manuscript.

## Abbreviations

PSS:NICU, parental stressor scale: neonatal intensive care unit; NICU, neonatal intensive care unit.
